# The characteristics and treatment strategy for transolecranon fracture-dislocation of the elbow in children: a retrospective study

**DOI:** 10.1186/s12891-022-05249-1

**Published:** 2022-03-29

**Authors:** Fei Su, Chuan Sun, Bing Wang, Min Li, Ji Ning Qu, Ya Ting Yang, Yong Tao Wu, Qiang Jie

**Affiliations:** 1 Pediatric Orthopaedic Hospital, Honghui Hospital, Xi’an Jiaotong University, Xi’an, 710000 China; 2 Research Center for Skeletal Developmental Deformity and Injury RepairSchool of Life Science and Medicine, Northwest University, 229 Taibai North Road, Xi’an, 710000 China; 3 Clinincal Research Center for Pediactric Skeletal Deformity and Injury of Shaanxi Province, Xi’an, 710000 China

**Keywords:** Ulnar fractures, Dislocations, Transolecranon, Fixation, Pathological characteristics, Children

## Abstract

**Background:**

Transolecranon fracture-dislocation of the elbow is rarely seen in children. The purpose of this retrospective study was to discuss the pathological characteristics and treatment strategy for this injury in children.

**Methods:**

From October 2016 to March 2019, 15 patients seen and treated at our institutions for transolecranon fracture-dislocation of the elbow were identified, and their medical records and radiographs were reviewed retrospectively. There were 11 boys and 4 girls, with an average age of 8.3 years (from 5 to 14 years). The left arm was involved in 10 cases, and the right arm was involved in 5 cases. Type I (simple fracture) was found in 11 cases, and type II (comminuted fracture) was found in 4 cases, 3 of which with coronoid process involved. Closed reduction was successful under local anaesthesia in 14 cases but failed in 1 case. In 11 patients with type I fractures, 10 received fixation of Kirschner wire and tension band, and one patient underwent bone plate fixation. In 4 patients with comminuted fractures (type II), internal fixation was performed with Kirschner wires combined with reconstruction plates.

**Results:**

The 15 patients were followed up for 24 to 48 months (average, 30.2 months). The final evaluation showed fine anatomical relationship of the elbow in all with no complications observed. Failure of internal fixation did not occur in any patient. The fractures acquired bony union in all patients after 8 to 12 weeks (average, 9.6 weeks). The therapeutic efficacy was evaluated at the final follow-up by the Mayo elbow performance score (MEPS) as excellent in 11 cases, good in 3 cases and fair in one case.

**Conclusions:**

As a type of complicated fracture-dislocation of the elbow, the transolecranon fracture-dislocation is rare in children. The fracture is mainly simple type. Treatment options depend on the type of fracture-dislocation. Only anatomical reduction of the olecranon fracture and restoration of a normal trochlear notch can lead to a stable humeroradial joint and good clinical efficacy.

## Background

In 1974, Biga first reported transolecranon fracture-dislocation of the elbow in adults [1]. However, this injury has been rarely reported in children since that report. At present, only 7 cases in 4 articles are available [2–5]. Transolecranon fracture-dislocation of the elbow in children is a complex fracture-dislocation that has unique pathological characteristics and treatment principles. Due to the difficulty of initial diagnosis, it is easily misdiagnosed as a type I Monteggia fracture, elbow varus-rotation instability or anterior dislocation of the elbow with olecranon fracture. Moreover, improper treatment may lead to forearm compartment syndrome, elbow instability, limited range of motion or even stiffness of the elbow, which may seriously affect the function of the affected limb [6]. In recent years, the incidence of complex elbow fractures and dislocations in children has gradually increased. Therefore, it is worth paying attention to avoiding misdiagnosis and improper treatment [7]. We reviewed 15 cases diagnosed as transolecranon fracture-dislocation of the elbow in children in our department from October 2016 to March 2019. By summarizing and analysing the clinical characteristics, diagnosis and treatment of the disease, we hope it will have clinical significance and avoid misdiagnosis and improper treatment.

## Methods

This is a single-institution retrospective study describing the treatment and outcomes of transolecranon fracture-dislocation of the elbow in children. The hospital database was searched in children who were admitted to our hospital between October 2016 and March 2019. This study was approved by the ethics committee of Honghui Hospital, Xi’an Jiaotong University. All guardians of the minors provided written informed consent prior to participation in the study. Inclusion criteria: (1) Children aged 0–15 years; (2) Children with dislocation of the elbow; (3) Children with fracture of the olecranon of ulna; (4) Children could be followed up completely. Exclusion criteria: (1) Children older than 15 years; (2) Children with multiple fractures; (3) Children with open fractures; (4) Children need surgical exploration because of neurovascular injuries; (5) Children with incomplete clinical data.

According to the literature, the morphology of olecranon fracture in transolecranon fracture-dislocation in adults is classified into type I (simple type) and type II (comminuted type) [8]. The type I was characterized by transverse or oblique fracture, while type II was characterized by comminuted fracture alone, or accompanied by an associated injury.

All patients were treated with closed reduction and the application of plaster under local anaesthesia in the emergency room. After reduction, X-ray were performed to evaluate the fracture and dislocation. For those with satisfactory dislocation reduction, only open reduction and internal fixation were performed for fractures. If closed reduction failed, open exploration, reduction and internal fixation was performed.

Open reduction was performed after induction of general anaesthesia. A midline longitudinal dorsal incision was made from the supracondyle of the humerus to the distal 3 to 4 cm part of the fracture. The skin and subcutaneous tissue were cut in layers, the anconeus muscle was separated with subperiosteal dissection, and the fracture ends were revealed. The pathological characteristics of fractures and dislocations were then evaluated. If the fracture of the olecranon was transverse or short oblique, internal fixation with tension-band application was used with 2–3 K-wires and single tension-band wiring after reduction. When an olecranon fracture was comminuted or combined with a bone defect, it was fixed with K-wires first after reduction, and then fixed with a reconstruction plate if C-arm fluoroscopy confirmed a satisfactory reduction and normal brachioradial relationship. After confirming satisfactory reduction of fracture-dislocation and reliable internal fixation by fluoroscopy, the wound was sutured. The elbow was fixed with a plaster bracket for 3 weeks postoperatively, and functional therapy was applied after the removal of plaster.

At 1, 2, 6 and 12 months post-op, patients were contacted by phone and returned for follow-up examination and radiographic evaluation under a protocol approved by the ethics committee. Anteroposterior and lateral radiographs were obtained to assess bony union, dislocation, ischaemic necrosis of the trochlea, early closure of the epiphysis, and heterotopic ossification. The hardware were removed after fracture healing.

Clinical examination included assessment of elbow stability and active range of motion. The therapeutic efficacy was evaluated at the final follow-up by the Mayo elbow performance score (MEPS). According to the MEPS, 90–100 points are considered excellent, 75–89 points as good, 60–74 points as fair and 0–59 points as poor results.

SPSS 23.0 software was used to process the data and measurement data. Continuous variables are expressed as mean ± standard deviation, and dichotomous variables are reported as raw numbers or percentages. Differences were analyzed using Fisher’s exact test for categorical variables and the Mann–Whitney U test for continuous variables. *p*-values < 0.05 were considered statistically significant.

## Results

Patient demographics are summarized in Table 1. The mean follow-up was 30.2 (range, 24 to 48 months). All injuries were unilateral, and there were 11 boys and 4 girls with an average age of 8.3 years (range, 5 to 14 years). The left, non-dominant limb was involved in 10 cases, and the right, dominant extremity was involved in 5 cases. The causes of injury included falling from a scooter in 7 cases, falling from horizontal bars in 3 cases, falling from stairs in 2 cases, cycling in 2 cases and traffic accident in 1 case. All were closed injury, with a mean of 9.2 h (ranging from 2 to 24 h) after the injury to consultation.

**Table 1:** The characteristics, treatment strategy and outcome for the transolecranon fracture-dislocation in children

Case	Gender	Age	Fracture type	Combined injury	Treatment and selection of internal fixation	Efficacy (MEPS)
1	M	9	Simple (transverse fracture)	No	OR, K-wires and tension-band wiring	Excellent
2	M	7	Simple (transverse fracture)	No	OR, K-wires and tension-band wiring	Excellent
3	M	5	Simple (transverse fracture)	No	OR, K-wires and tension-band wiring	Excellent
4	M	14	Simple (transverse fracture)	Medial epicondyle of humerus	OR, K-wires and tension-band wiring	Good
5	F	8	Simple (transverse fracture)	No	OR, K-wires and tension-band wiring	Good
6	M	6	Simple (transverse fracture)	No	OR, K-wires and tension-band wiring	Excellent
7	F	9	Simple (transverse fracture)	No	OR, K-wires and tension-band wiring	Excellent
8	M	11	Comminuted	No	OR, K-wires and reconstruction plate	Excellent
9	M	13	Comminuted	Radial neck Coronoid process	OR, K-wires and reconstruction plate	Fair
10	M	14	Comminuted	Coronoid process	OR, K-wires and reconstruction plate	Excellent
11	F	12	Comminuted	Coronoid process	OR, K-wires and reconstruction plate	Good
12	F	14	Simple (transverse fracture)	No	OR, K-wires and tension-band wiring	Excellent
13	M	7	Simple (transverse fracture)	No	OR, K-wires and tension-band wiring	Excellent
14	M	8	Simple (transverse fracture)	No	OR, K-wires and tension-band wiring	Excellent
15	M	10	Simple (short oblique fracture)	No	OR, reconstruction plate	Excellent

The clinical manifestations were significant pain with obvious deformity of the elbow and significant swelling. The patients could not bend and stretch the elbow actively. When the elbow was moved passively, the movement was limited due to pain. The skin and neurovascular status were intact, and the passive pull pain test was negative.

In this study, we found 11 patients with type I (simple type) proximal ulna fractures and 4 patients with type II (comminuted type). Three of these were associated with coronoid fracture. Closed reduction was successful under local anaesthesia in 14 cases but failed in 1 case. In 11 patients with type I (simple type) fractures, 10 received fixation of K-wires and tension band, and one patient with short oblique fracture underwent bone plate fixation. In 4 patients with comminuted fractures, internal fixation was performed with K-wires combined with reconstruction plate. All cases acquired bony union after 8 to 12 weeks (average, 9.2 weeks) postoperation. The time of hardware removal was 3 to 7 months (average, 4.2 months). At the last follow-up, the elbow joint was well matched in anatomy in all patients, with no recurrence of dislocation, no trochlear necrosis and early closure of the epiphysis. The therapeutic efficacy was evaluated at the final follow-up by the Mayo elbow performance score (MEPS) as excellent in 11 cases, as good in 3 cases and as fair in one case. At the last follow-up, a limitation of 10° of elbow extension has been recorded in this child, but with no impact on normal function. The data are summarized in Table 1.

## Discussion

Transolecranon fracture-dislocation of the elbow is common in adults but rare in children. There is no report on the incidence of children at present, and most cases are caused by high energy injuries [9]. Most reports describe this injury occurring when a high-energy is applied directly to the proximal dorsal forearm with the obstruction of the trochlea to olecranon, which causes olecranon fracture associated with anterior dislocation of the proximal ulna and radius together [10]. However, it has been reported that when the elbow is in a hyperextended position, the proximal ulna acts against the olecranon fossa, which results in olecranon fracture. The further sustained effect of external force results in anterior dislocation of the distal complex of fracture [4]. Transolecranon fracture-dislocation of the elbow is a type of anterior dislocation in which compromise of the ulnohumeral articulation occurs through an complex injury to the proximal ulna, while the annular ligament and the proximal radioulnar joint remain intact [11].

**Fig. 1:**
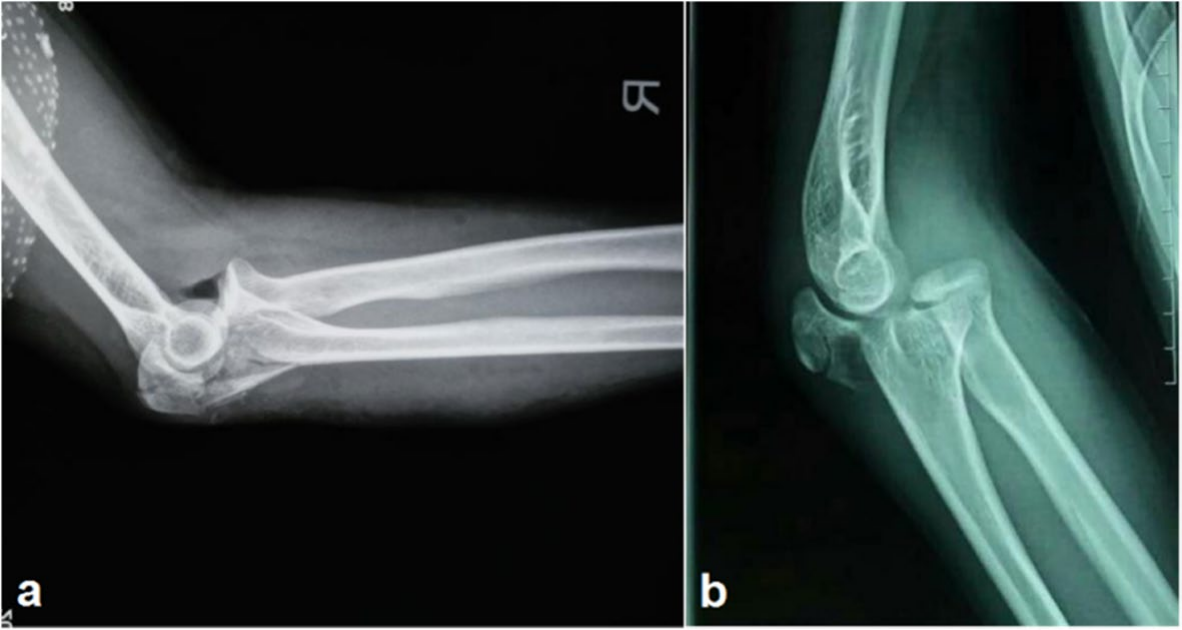
Lateral radiograph of the elbow in a 42-year-old-male patient with comminuted fracture of proximal ulna, combined with fracture of coronoid process and anterior dislocation of distal complex of fracture (**a**); Lateral radiograph of the elbow in a 14-year-old boy (case 4) with transverse fracture of olecranon, small proximal fracture fragment, combined with anterior dislocation of distal complex of fracture, no involvement of coronoid process (**b**)

In adults, fractures of the olecranon or proximal ulna are often serious. Type II fractures (comminuted type) are common, and most patients have coronoid process fractures. Therefore, stable anatomic reduction of the proximal ulna in adults with transolecranon fracture-dislocation is complex and difficult. Fixation of K-wires combined with reconstruction plate is often required, and bone grafts may sometimes be needed [12]. But in our cases, 11 patients suffered simple type of proximal ulna fracture. And 4 patients had comminuted type, 3 of them with coronoid process fracture. This difference may be related to children's periosteum hypertrophy, bone flexibility and relative relaxation of the elbow ligament, however, the situation is completely different in adults (shown in Fig 1). Therefore, we believe that most transolecranon fracture-dislocations in children are simple type and not as complicated and serious as those in adults. This conjecture has not been reported before.

In children, transolecranon fracture-dislocation is easily misdiagnosed as a type I Monteggia fracture and divergent anterior dislocation of the elbow with olecranon fracture. The characteristic Monteggia lesions are fracture-dislocations of the forearm, and the defining lesion is a dislocation of the proximal radioulnar joint. In addition, Monteggia’s classification of fracture-dislocations does not include ulnohumeral articulation. For treatment of these fractures, it is necessary to restore the normal alignment of the proximal ulna, which is fundamentally different from that of transolecranon fracture-dislocation. In this study, transolecranon fracture-dislocation of the elbow was a type of anterior dislocation of the elbow, while the proximal radioulnar joint remained intact. This is also different from Suzuki's report of a case of divergent anterior dislocation of the elbow with olecranon fracture [13]. This report described olecranon fracture of the ulna and anterior dislocation of the elbow joint with dislocation of the upper radioulnar joint and complete rupture of the annular ligament [13]. Therefore, the essential difference between transolecranon fracture-dislocation and type I Monteggia fracture and divergent anterior dislocation of the elbow with olecranon fracture is that the upper radioulnar joint is intact and stable. So, once the olecranon fracture has gained anatomic reduction, the brachioradial joint can be automatically reduced and matched, and the elbow can also be stabilized.

**Fig. 2:**
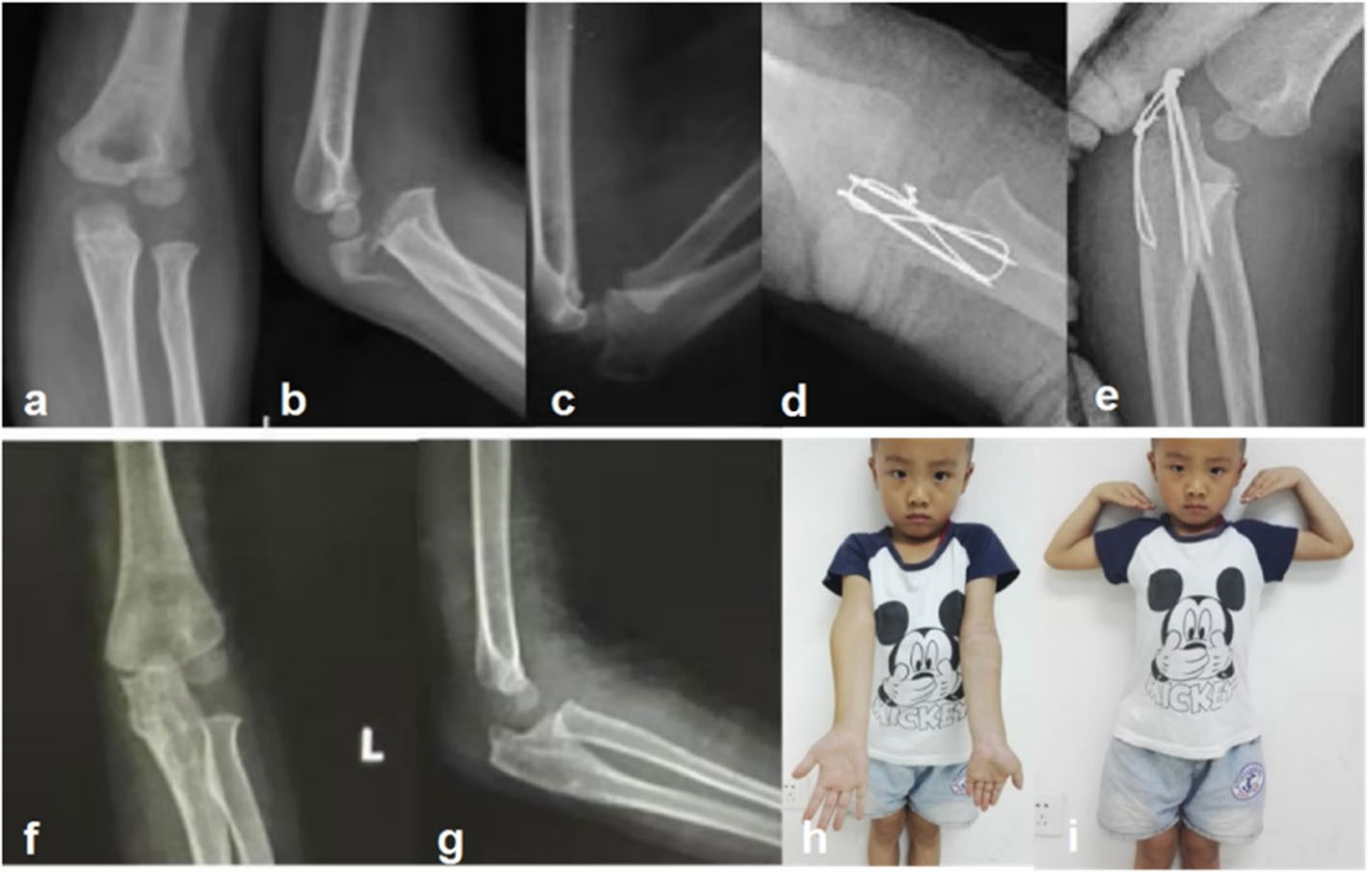
A 5-year-old boy (Case 3) with transverse fracture of olecranon, anterior dislocation of distal fracture and proximal radius (**a**, **b**); The fracture-dislocation was generally corrected after closed reduction (**c**); Open reduction and internal fixation of olecranon fracture, K-wire tension band fixation and correction of elbow dislocation were performed (**d**,**e**); The internal fixator was removed, and the fracture healed well and the elbow joint matched well (**f**, **g**); Elbow flexion and extension were normal (**h,** **i**)

**Fig. 3:**
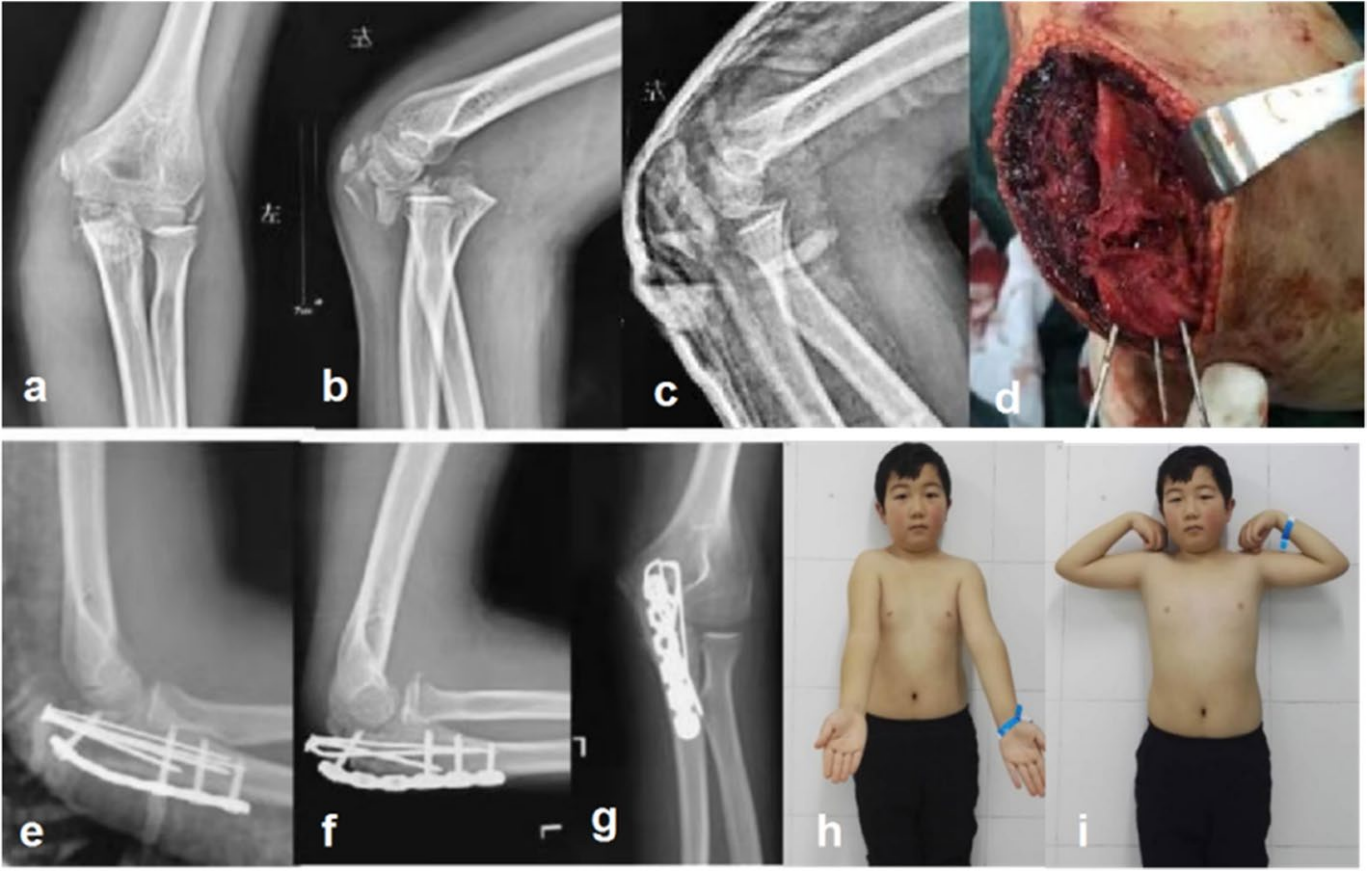
An 11-year-old boy (Case 8) with an olecranon comminuted fracture that does not involve the coronoid process and anterior dislocation of the distal complex of the fracture (**a**, **b**). Fracture-dislocation was generally corrected after closed reduction (**c**). K-wire was used for temporary fixation after anatomical reduction of the olecranon articular surface of the ulna. Part of the bone defect at the back of the metaphysis of the ulna was found, so it was fixed with K-wires combined with a reconstruction plate. (**d**, **e**); At the end of follow-up, the fracture healed well, and the elbow joint matching was satisfactory (**f**, **g**); the elbow function was normal (**h**, **i**)

The dislocation of elbow in most children can been successfully reduced with closed reduction under local anaesthesia, based on the force of the injury mechanism. After reduction of dislocation, only a midline longitudinal dorsal incision was used for open reduction and internal fixation for olecranon fractures, which is conducive to early functional training of the elbow. If closed reduction failed, open exploration, reduction and internal fixation can be performed. The ulnar trochlear notch is 180° around the trochlea. Its integrity is important for the stability of elbow [14]. The interlocking of the olecranon and the coronoid process in their corresponding fossae on the distal aspect of the humerus provides additional stability at the extremes of ulno-humeral motion. Therefore, ulnar olecranon fractures should be anatomically reduced, especially in patients with coronoid process fractures, to restore the normal depth and width of the trochlear notch as much as possible [15]. If not, it will lead to instability or subluxation of the brachioradialis joint. The failure of treatment is due to improper treatment of the coronoid process fracture or incomplete restoration of the normal anatomical structure of the proximal ulna [16]. Olecranon fractures in children are usually less serious than those in adults, and these fractures in children are most often simple. Corradin used a cannulated screw to fix the olecranon fracture especially in older patients who did not have a comminuted fracture, with good effects and less complications [17]. However, considering the damage to the epiphysis and epiphyseal growth plate, we did not choose this fixation method. Therefore, reconstruction by smooth K-wires and tension bands is relatively easy and safe, without the combination of reconstruction plate. In addition, as proximal fracture fragment is small, the use of reconstruction plates may damage the epiphysis of the olecranon. However, K-wires combined with plate fixation is still needed in children with comminuted fracture. In our cases with simple type fractures, 10 patients received tension band with K-wire fixation(shown in Fig. 2), and one patient with a short oblique fracture underwent bone plate fixation. In 4 patients with comminuted fractures, internal fixation was K-wires combined with reconstruction plates (shown in Fig. 3).

The elbow was fixed with a plaster bracket for 3 weeks postoperation and function training was started after plaster removal. Patients were followed for an average of 30.2 months (range, 24–48 months). At the last follow-up, the therapeutic efficacy was evaluated at the final follow-up by the Mayo elbow performance score (MEPS) as excellent in 11 cases, as good in 3 cases and as fair in one case. At the last follow-up, a limitation of 10° of elbow extension has been recorded in this child, but with no impact on normal function.

There are many limitations to this study. First, this is a retrospective analysis, and the number of cases of each type is small, therefore we were not able to carry out statistical comparative analysis. In addition, the follow-up time of some patients was short, so whether there would be later development of complications is still difficult to predict.

## Conclusions

In summary, transolecranon fracture-dislocation of the elbow in children is a rare complex fracture-dislocation. Transolecranon fracture-dislocation of the elbow in children is mainly simple. Treatment options depend on the type of fracture and dislocation. Only anatomical reduction of olecranon fractures and restoration of normal trochlear notches can obtain stable brachioradialis joints and achieve better clinical results.

## Data Availability

The data and materials are available from the corresponding author on reasonable request. All authors share their raw data, and we summarize it in Table 1.

## Abbreviations

MEPS: Mayo elbow performance score
CT: Computed tomography
M: Male
F: Female
OR: Open reduction
